# Hypofractionated radiation therapy comparing a standard radiotherapy schedule (over 3 weeks) with a novel 1-week schedule in adjuvant breast cancer: an open-label randomized controlled study (HYPORT-Adjuvant)—study protocol for a multicentre, randomized phase III trial

**DOI:** 10.1186/s13063-020-04751-y

**Published:** 2020-09-30

**Authors:** Sanjoy Chatterjee, Santam Chakraborty, HYPORT Adjuvant Author Group, Santam Chakraborty, Selvamani Backianathan, Punita Lal, Subhash Gupta, Rosina Ahmed, Shagun Misra, Patricia Solomon, Rajesh Balakrishan, Debashree Guha, K. J. Maria Das, Anurupa Mahata, Samar Mandal, Abha Kumari, Henry Finlay Godson, Sandip Ganguly, Debdeep Dey, Sanjoy Chatterjee

**Affiliations:** Department of Radiation Oncology, Tata Medical Center, Kolkata, India

**Keywords:** Breast cancer, Hypofractionation, Adjuvant radiotherapy, One-week radiotherapy, Non-inferiority trial

## Abstract

**Background:**

Hypofractionated radiotherapy is the current standard for adjuvant radiotherapy across many centres. Further hypofractionation may be possible but remains to be investigated in non-Caucasian populations with more advanced disease, with a higher proportion of patients requiring mastectomy as well as tumour bed boost. We are reporting the design of randomized controlled trial testing the hypothesis that a 1-week (5 fractions) regimen of radiotherapy will be non-inferior to a standard 3-week (15 fractions) schedule.

**Methods:**

We describe a multicentre, randomized controlled trial recruiting patients at large academic centres across India. Patients without distant metastases who merit adjuvant radiotherapy will be eligible for inclusion in the study. Patients in the control arm will receive adjuvant radiotherapy to the breast or chest wall (with/without regional nodes) to a dose of 40 Gy/15 fractions/3 weeks, while those in the experimental arm will receive a dose of 26 Gy/5 fractions/1 week (to the same volume). The use of a simultaneous integrated boost (dose of 8 Gy and 6 Gy, respectively) is allowed in patients who have undergone breast conservation. A sample size of 2100 patients provides an 80% power to detect a non-inferiority of 3% in the 5-year locoregional recurrence rate with a one-sided type I error of 2.5%, assuming that the locoregional recurrence rate in the control arm is 5% at 5 years (corresponding to a hazard ratio of 1.63). Patients will be recruited over a period of 5 years and followed up for a further 5 years thereafter.

**Discussion:**

If a five-fraction regimen of breast cancer is proven to be non-inferior, this will result in a significant improvement in the access to radiotherapy, as well as reduced costs of treatment. The trial gives an opportunity to standardize and quality-assure radiotherapy practices across the nation at the same time. Along with the results of the FAST-Forward trial, the safety of this intervention in advanced node-positive disease requiring regional nodal radiation will be established.

**Trial registration:**

The trial has been registered at the Clinical Trial Registry of India (CTRI) vide registration number: CTRI/2018/12/016816 (December 31, 2018) as well as the ClinicalTrial.gov website at NCT03788213 (December 28, 2018).

## Administrative information

**Table:** 

**Title{1}**	Hypofractionated radiation therapy comparing a standard radiotherapy schedule (over 3 weeks) with a novel 1-week schedule in adjuvant breast cancer: an open-label randomized controlled study (HYPORT-Adjuvant)—study protocol for a multicentre, randomized phase III trial
**Trial Registration{2a and 2b}**	The trial has been registered at the Clinical Trial Registry of India (CTRI) vide registration number: CTRI/2018/12/016816 as well as the ClinicalTrial.gov website at NCT03788213.
**Protocol version {3}**	6.0 dated 8th July 2019
**Funding {4}**	Intramural funding from Tata Medical Center Extramural funding from Nag Foundation Additional extramural funding sought from National Cancer Grid of India
**Author Details {5a}**	1. Dr Sanjoy Chatterjee, Department of Radiation Oncology, Tata Medical Center, Kolkata, West Bengal, India, 700,156 2. Dr. Santam Chakraborty, Department of Radiation Oncology, Tata Medical Center, Kolkata, West Bengal, India, 700,156 3. HYPORT Adjuvant Author Group
**Name and Contact Information of Trial Sponsor {5b}**	Tata Medical Center, 14 MAR (E-W), New Town, Action Area III, Kolkata, West Bengal 700,156. Phone: 91-3366,057,402
**Role of Sponsor {5c}**	The Sponsor (Tata Medical Center) is responsible for the trial conduct, reporting and oversight under the guidance of the Institutional Review Board. The trial design was done in collaboration with the other institutes mentioned above. Extramural funding has been obtained from the Nag Foundation for an acute toxicity substudy. The Nag foundation has no role in the study design as the collection, management, analysis, and interpretation of data; writing of the report; and the decision to submit the report for publication

## Introduction

### Background and rationale {6a}

#### Breast cancer burden and role of radiotherapy

Breast cancer is the commonest cancer occurring in Indian females and also the cause of the greatest degree of cancer-related mortality and disability amongst our population [1]. Adjuvant radiation therapy reduces the 10-year locoregional recurrence rates (LRR) from 35 to 19.6%. This advantage translates into an improved breast cancer survival (25.2% versus 21.4%) at 15 years in patients undergoing breast conservation [2]. A similar benefit is seen in patients who have undergone mastectomy [3].

Unfortunately, despite the important role that adjuvant radiotherapy plays in the management of breast cancer, access to radiotherapy is a limiting factor impacting control rates [4, 5]. It is known that access to radiotherapy impacts the type of surgery performed, and improving accessibility increases uptake of breast conservation therapy [6]. One of the important methods by which access to scarce radiotherapy can be improved in resource-limited settings is by adopting a shorter hypofractionated course of radiotherapy where the radiation is delivered in higher daily doses (> 2 Gy) for a fewer number of fractions.

#### Radiobiological basis of hypofractionation in breast cancers

Breast cancer is one of the few malignancies where the fraction sensitivity is high, which in turn implies that use of a higher dose per fraction is associated with lower cell survival. The “recovery exponent” for breast cancer was quite similar to that of the normal skin, making protracted fractionation ineffective in these tumours [7]. Based on this data, a randomized trial investigating two experimental schedules of 13 fraction (39 Gy and 42.9 Gy) against the standard arm of 50 Gy in 25 fractions was completed at the Royal Marsden Hospital and Gloucestershire Oncology Center in 1998. The results of this trial were used to derive point estimates of the α/β ratio for both late normal tissue reactions (which ranged between 3 and 4 Gy) [8], as well as for tumour control (which was 4.1 Gy) [9]. Subsequently, when the results of the Canadian hypofractionation trial [10] and the two START trials [11] were combined, it became apparent that the point estimate for the α/β ratio of tumour control was 3.5 (95% CI 1.2–5.7) [11].

#### Hypofractionation in breast cancer

Following the results of the RMH/GOC trial [12], several subsequent trials investigated the role of hypofractionated radiotherapy with a dose reduction delivered over 3 weeks in breast cancers. A meta-analysis of these trials has shown that hypofractionated radiotherapy is similar to conventionally fractionated radiotherapy in terms of locoregional control, disease-free survival and overall survival [13]. Additionally, it is associated with reduced acute toxicity and similar late toxicity. Taken together, these trials have investigated more than 8000 women with breast cancers [12] and have established the safety of this approach.

#### Further hypofractionation

While 3 weeks of radiation is a reduction in the total time, further hypofractionation is biologically plausible based on the fraction sensitivity of mammary carcinoma. Courdi et al. had reported a case series of elderly unfit patients who underwent definitive radiotherapy with once-weekly hypofractionated radiotherapy of 6.5 Gy for 5 fractions followed by a boost of 6.5 Gy for 1–3 fractions to the tumour site along with endocrine therapy [14]. They reported a 5-year local progression-free survival of 78%. Subsequently, the UK FAST trial tested two 5 fractions weekly regimen of hypofractionated radiotherapy (28.5 Gy or 30 Gy) against the standard arm of 50 Gy [15]. The acute toxicity results, as well as the results of the photographic assessment of breast appearance, showed that the regimen of 28.5 Gy was equivalent to 30 Gy. Locoregional control was similar in both arms, with no difference in cosmesis or late toxicity profile. Additional studies investigating the safety and efficacy of a once-weekly regimen have demonstrated similar results [16, 17].

Based on the results of the FAST trial, and as there was a possibility of gain in the tumour control with a reduction in the overall treatment time, the FAST-Forward trial was designed to evaluate two schedules of 5 fraction radiotherapy delivered in 1 week against the standard arm of 15 fraction radiotherapy delivered in 3 weeks. The results of acute skin toxicity originating from this protocol have been reported. The regimen of 26 Gy delivered in 5 fractions is associated with a grade 3 RTOG toxicity of 5.8%, versus 13.6% for the control arm [18]. None of the patients had a CTCAE grade 3 toxicity in either of the arms [18].

#### Simultaneous integrated boost (SIB)

An important contributor to the overall treatment time is the tumour bed boost in patients receiving adjuvant radiotherapy after breast conservation surgery. The results of the seminal European Organisation for Research and Treatment of Cancer (EORTC) trial have demonstrated that boost significantly improves the local control after breast conservation surgery [19].

We reviewed the published literature in PubMed using the search terms SIB OR Simultaneous Integrated Boost OR SMART OR Concomitant Boost AND Breast cancer. Five hundred twenty-one articles were found, of which 83 articles were found to be relevant. Ten of these studies are prospective trials, and medium to long-term outcomes have been reported for seven. These are summarized below (Table 1).

**Table 1:** Table of salient details of major prospective trials investigating simultaneous integrated boost in breast cancer

Author (year)	***N***	RCT	Dose fractionation	BED (Gy3)	FU	Local control	Late toxicity
Franco (2014), Turin, Italy	82	No	45 + 5/20#	91.67	12	100	0
Parjis (2012) Brussels, Belgium	70	Yes	42 + 9/15#	108.80	12	NA	25% fibrosis (Gr1)
Mandal (2017), Delhi, India	10	No	40.5 + 7.5/15#	99.20	24	100	NA
Shin (2016), New York, USA	45	No	40.5 + 7.5/15#	99.20	36	100	4% lymphedema (G2), 2% skin retraction (G3)
De Rose (2016), Milan, Italy	144	No	40.5 + 7.5/15#	99.20	37	100	NA
Cooper (2016), New York, USA	400	Yes	40.5 + 7.5/15#	99.20	45	99%	5.9% fibrosis (G2)
Cante (2017), Turin, Italy	178	No	45 + 5/25#	83.33	117	97.3	7% fibrosis (G2),

In addition, other studies have reported on the acute toxicities and patient-reported outcome. One randomized controlled trial reported by Paelink et al. demonstrated no increase in moist desquamation after SIB in a randomized trial of 167 patients [20]. Another randomized controlled trial compared hypofractionated radiotherapy with SIB to conventionally fractionated radiotherapy with sequential boost. The shorter regime resulted in better recovery of functional status and faster recovery from fatigue [21].

The results from the IMPORT-High trial as presented at SABCS 2018 demonstrate that SIB (48 Gy) was associated with similar rates of moderate to severe breast induration, shrinkage, distortion and change in photographic appearance as a sequential boost (40 Gy + 16 Gy). A mild to marked change in photographic appearance was observed in 12 patients undergoing SIB versus 35 patients undergoing sequential boost (*p* = 0.03) [22].

#### The rationale of this trial

If the results of FAST-Forward trial are positive, 1-week whole breast radiotherapy may be adopted as a standard of care in the UK. However, this study has been conducted in a predominantly screen-detected Caucasian population. Advanced cancers and also younger patients with triple-negative cancers are under-represented. In our population, the majority of patients need a tumour bed boost, and thus, in order to have a truly 1-week course of radiotherapy, the tumour bed boost needs to be delivered as a SIB.

Hence, a trial investigating a 1-week whole breast radiotherapy schedule with a SIB for the tumour bed needs to be conducted in our setting. We therefore plan to integrate a FAST-Forward like 1-week radiotherapy schedule with a SIB with appropriate dose reduction as done in the IMPORT-High trial.

### Objectives {7}

We hypothesize that the locoregional recurrence rate after a 1-week (5 fractions) course of adjuvant radiotherapy will be non-inferior to the standard 3-week course (15 fractions). The primary objective is to compare the cumulative incidence of patients experiencing a locoregional recurrence by 5 years between 1-week and three-week course of radiotherapy. Based on an audit of the outcomes of patients treated at our centre, the cumulative proportion of the locoregional recurrence rate in the control arm is expected to be 5% at 5 years. A non-inferiority margin of 3% would be clinically acceptable in this setting.

The secondary objectives are to compare:

1. Invasive disease-free survival, defined as the duration of time from randomization to disease recurrence, death due to any cause or any second invasive malignancy at 5 years
2. Overall survival defined as the duration of survival from the randomization at 5 years.
3. Rate of CTCAE (version 5.0) grade 3 or more late radiation-related adverse events.
4. The proportion of patients with health-related quality of life (QoL) similar to or better than the baseline at 12 months.

### Trial design {8}

**Fig. 1:**
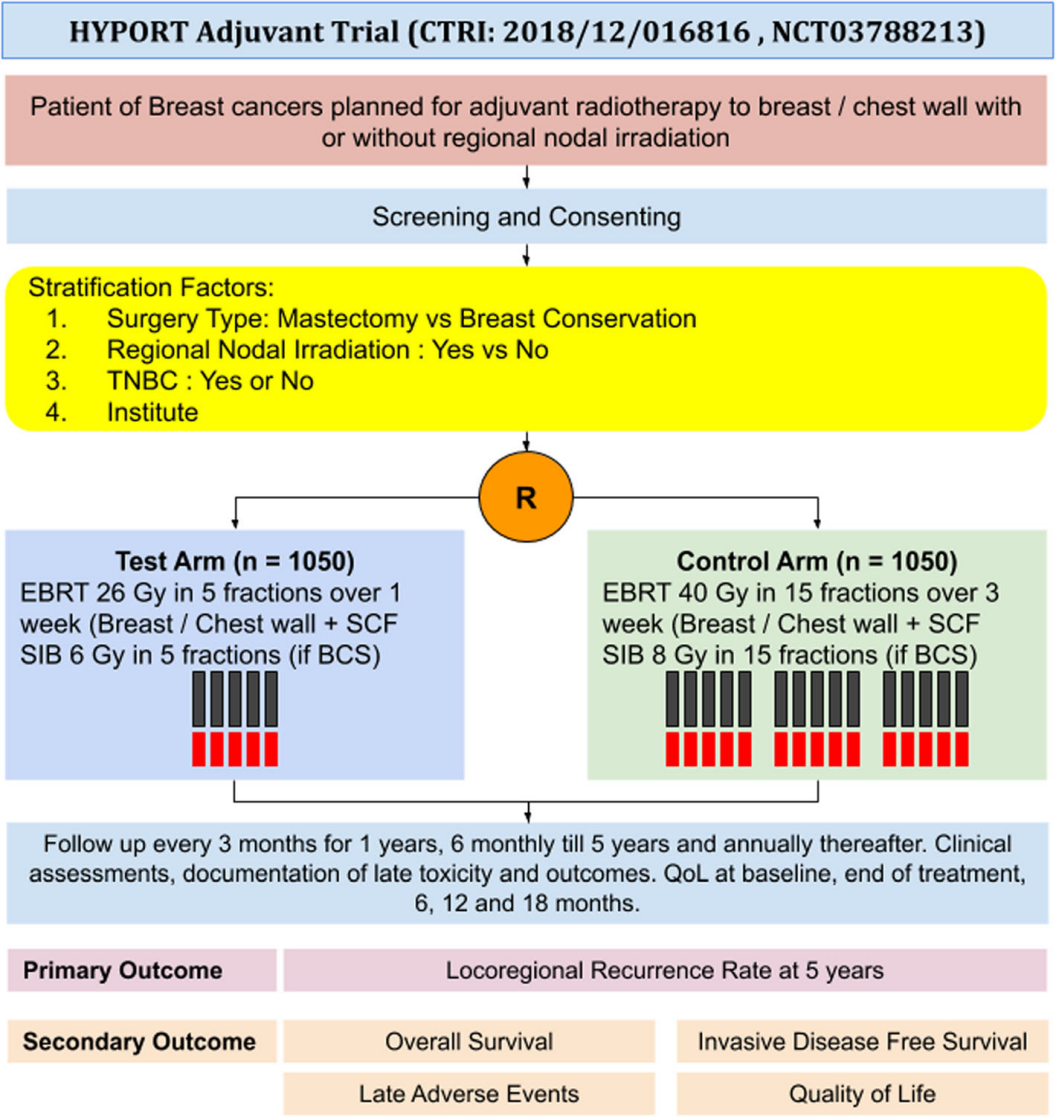
HYPORT-Adjuvant trial schema

The HYPORT-Adjuvant trial is an investigator-driven, open-label, parallel-group, two-arm, non-inferiority, randomized controlled trial of 10-year total duration. Patients will be accrued for 5 years in this trial and allocated equally in the two arms. Figure 1 shows the trial schema.

## Methods: participants, interventions and outcomes

### Study setting {9}

The trial is supported currently by intramural funding from Tata Medical Center and has received extramural funding from the NAG foundation. The study has been initiated at the Department of Radiation Oncology at Tata Medical Center. Site initiation has been done at CMC Vellore and SGPGI Lucknow. Combining all three centres, approximately 100 patients annually undergo adjuvant radiotherapy. As the eligibility criteria are very inclusive we expect that most breast cancer patients requiring adjuvant radiotherapy will be eligible for inclusion in this study. Other large academic centres treating a substantial number of breast cancer patients will be approached for participation in the future.

### Eligibility criteria {10}

#### Patient eligibility criteria

Inclusion criteria are deliberately kept non-restrictive so that the results can be generalizable to the Indian population of breast cancer patients. Patients who have undergone curative-intent surgery in the form of a mastectomy or breast conservation surgery with clear margins for non-metastatic invasive breast cancers are eligible for this trial. An adequate axillary clearance or an appropriate axillary staging procedure like sentinel lymph node biopsy should have been performed. All patients undergoing breast conservation or neoadjuvant chemotherapy will be eligible. Patients will be eligible for inclusion after mastectomy if they have the following characteristics:

1. T3–T4 tumours
2. > 3 axillary lymph nodes
3. T0–T2 tumour with 0–3 axillary lymph nodes with a Cambridge Score of 3 or more [23].

The primary exclusion criteria are patients with carcinoma in situ, non-epithelial breast primaries and metaplastic carcinomas. Patients with a residual internal mammary node or supraclavicular fossa node prior to radiotherapy will also be excluded as they may be eligible for an additional boost to the residual node. Patients with prior radiotherapy to the breast or those planned for concurrent chemotherapy will also be excluded.

#### Trial centre requirements

High-volume centres treating more than 150 breast cancer patients annually with facilities for surgery, radiotherapy and chemotherapy as well as a functional pathology and radiology services will be required. Ideally, there should be a dedicated multi-disciplinary team comprising of surgeons, clinical/radiation oncologists, medical oncologists, pathologists and radiologists treating breast cancer. The centre should report mammograms using the Breast Imaging Reporting and Data (BIRAD) system. Breast pathology services should have accredited services for evaluation of oestrogen receptor, progesterone receptor and Her2neu receptor status with immunohistochemistry.

For this study, breast cancer patients must be simulated through a volumetric imaging technique. The trial centres must fulfil the trial quality assurance requirements as outlined in the Radiotherapy Quality Assurance (RTQA) document.

### Who will take informed consent {26a}

Informed consent will be obtained from the patient by the investigators or designated authorized person. Written informed consent will be obtained on the informed consent form.

### Additional consent provisions for collection and use of participant data and biological specimens {26b}

Biospecimen banking in the form of plasma and buffy coat will be done for consenting patients. A separate consent will be obtained by the investigators for this. The biobanking consent form used in TMC will be used for specimen biobanking in Tata Medical Center. For other centres, biobanking of biological specimens will be done at investigators’ discretion following institutional policy.

## Interventions

### Explanation for the choice of comparators {6b}

One-week hypofractionated radiotherapy is likely to become the standard for delivery of breast adjuvant radiotherapy if the results of the FAST-Forward trial support the primary objective. Hence, the current trial will also test a 1-week regimen. The 3-week course of radiotherapy used in the control arm has already demonstrated non-inferiority in terms of oncological outcomes in the START trials. Currently, this is the standard radiotherapy schedule used for most breast cancer patients in the participating centres.

### Intervention description {11a}

#### Overview

Patients will be randomized into two arms equally:

1. Control Arm: 40 Gy in 15 fractions over 3 weeks to the whole breast or chest wall. Patients undergoing breast conservation therapy will receive additional boost radiotherapy to the tumour bed. The supraclavicular fossa will be treated in patients with node-positive disease or those receiving neoadjuvant chemotherapy. IMC and axillary radiotherapy will be given as per the institutional policy. Boost radiotherapy will be delivered using SIB of 8 Gy in 15 fractions (or alternatively using a sequential boost of 12 Gy in 4 fractions)
2. Test Arm: 26 Gy in 5 fractions over 1 week to the whole breast or chest wall. Treatment volumes will be the same as the control arm. Additional boost will be delivered to patients who have undergone breast conservation. Boost radiotherapy will be delivered using SIB of 6 Gy in 5 fractions (or alternatively using a sequential boost of 12 Gy in 4 fractions)

#### Radiotherapy

Radiotherapy should be started within 12 weeks of the last date of surgery or last cycle of planned adjuvant chemotherapy. All patients would be treated on linear accelerators (6–15 MV energies). Radiotherapy planning would be performed on volumetric planning CT scans with a pre-defined simulation protocol. Patients with left-sided breast cancers should preferably receive cardiac sparing radiotherapy. Field-based planning for the breast and chest wall is allowed in the protocol where anatomical landmarks are identified and coverage ensured. This is the current practice in most of the centres in this trial.

The organs at risk to be delineated include the ipsilateral and contralateral lung, heart and the contralateral breast. In patients undergoing radiotherapy for left-sided breast, the left anterior descending artery may also be delineated [24]. The brachial plexus may be delineated in patients undergoing regional nodal radiation [25].

Tangential beams would be utilized for irradiating the breast or chest wall, while regional nodal radiation should be delivered using a technique that ensures no overlap of the field—in most patients, this would imply an enface field to cover the supraclavicular fossa (SCF) with a half beam block or a collimator-couch rotation-based technique. Dose will be prescribed at a point in the midplane for the breast and chest wall for tangents. For patients receiving SCF radiation, the dose will be prescribed at the depth of maximum dose (Dmax). In patients who have undergone breast conservation, boost will be delivered using the technique used in the institute. The use of a volumetric modulated arc therapy (VMAT)-based simultaneous integrated boost (SIB) for tumour bed irradiation is allowed. Inverse planned radiation is allowed for patients receiving radiation to the internal mammary nodal chain.

Dose in the target volume should be homogenous and use of a forward planned field in field IMRT technique is allowed. Alternatively, centres can specify the technique to be used for ensuring dose homogeneity. Individualized treatment planning will be done with a target to ensure that the breast/chest wall is covered with 95% of the prescribed dose and that the maximum dose does not exceed 107% of the prescribed dose. In patients undergoing VMAT simultaneous tumour bed boost (SIB), the dose to the boost target volume will be restricted to < 107% of the total dose (Table 2).

**Table 2:** The dose constraints to be used for plan evaluation

Volume	Standard arm			Test arm		
	Criteria	Mandatory	Optimal	Criteria	Mandatory	Optimal
BTV (only in patients receiving SIB)	D98	≥ 43.2 Gy	NA	D98	≥ 28.8 Gy	NA
	D95	≥ 45.6 Gy	NA	D95	≥ 30.4 Gy	NA
	D2	≤ 51.4 Gy	≤ 50.4 Gy	D2	≤ 34.2 Gy	≤ 33.6 Gy
	D0.03	≤ 52.8 Gy	≤ 51.4 Gy	D0.03	≤ 35.2 Gy	≤ 34.2 Gy
Heart	Mean	≤ 2.5 Gy	≤ 2 Gy	Mean	≤ 1.6 Gy	≤ 1.3 Gy
	V10	≤ 5%	≤ 3%	V7	≤ 5%	≤ 3%
	V2	≤ 30%	≤ 20%	V1.2	≤ 30%	≤ 20%
Ipsilateral lung	V12	≤ 30%	≤ 18%	V8	≤ 30%	≤ 18%

Treatment will be delivered 5 days a week treating one fraction a day. All fields will be treated together. Dosimetric quality assurance of all plans will be performed with point dose measurements along with fluence profile checks if IMRT is used. The centre will prespecify the imaging verification protocol to be used for their patients.

### Criteria for discontinuation and modifying allocated interventions {11b}

Breast cancer radiotherapy is not expected to result in substantial acute toxicity. Treatment should not be interrupted unless a patient has a grade III–IV CTCAE adverse event. After this treatment can be restarted once the toxicity has reduced to Grade I or less and if in the treating physician’s opinion it is safe to continue with treatment.

### Strategies to improve adhere to intervention {11c}

Patients will be counselled about the need to adhere to the treatment schedule. They will be reviewed weekly during treatment to discuss and manage any treatment-related adverse events they may be encountering. Any missed fraction would be informed to the treating team and attempts will be made to communicate with the patient to avoid this in future.

### Relevant concomitant care permitted or prohibited during the trial {11d}

No specific contraindications to any medication except for the receipt of concurrent chemotherapy. Patients may continue with planned endocrine therapy and anti-HER2 therapy during the course of radiotherapy.

### Provisions for post-trial care {30}

Routine follow-up care will be provided for all patients including appropriate clinical evaluation, adjuvant endocrine therapy, maintenance anti-HER2 therapy and chemotherapy as required. Compensation for trial-related injuries will be provided as per extant Indian laws after the review by the Institutional review board.

### Outcomes {12}

#### Primary outcome

The primary outcome of interest is the locoregional recurrence, which is defined as any invasive recurrence in the ipsilateral breast or chest wall or ipsilateral axillary, supraclavicular or internal mammary lymph nodes (ipsilateral lymph nodes level 1–4 and internal mammary nodes as defined by Offersen et al. [26]). Cumulative incidence of patients with locoregional recurrence at 5 years will be estimated and hazard ratio of the locoregional recurrence rate between the two groups will be reported.

#### Secondary outcomes

1. Overall survival: This is defined as the interval of time between the date of randomization to the date of death due to any cause. We will attempt to obtain the reason for death wherever feasible. The duration will be calculated using the actuarial method and patients who are alive at last follow up will be censored on the same date. Cumulative proportion of patients surviving at 5 years will be reported along with hazard ratio for the two arms.
2. Invasive disease-free survival: This is defined as the time from randomization to the time any recurrence (pre-invasive / invasive), distant metastases, death from any cause and second invasive primaries, including invasive neoplasms of the breast. Cumulative proportion of patients without invasive disease recurrence at 5 years will be reported along with hazard ratio for the two arms.
3. Adverse events (AE): The National Cancer Institute (NCI) Common Terminology Criteria for Adverse Events (CTCAE) version 5 will be used to classify and grade the severity of adverse events. Clinician reported AE grades would be collected in addition to patient-reported outcomes measures to be collected as a part of the assessment of quality of life. Adverse events due to radiotherapy (CTCAE 5.0) will be classified into the following categories:
  - Start of RT to 90 days after RT: Acute toxicity
  - > 90 days after RT: Late toxicity.
4. Quality of life: Quality of life will be assessed using self-administered EORTC QLQ C30 and the FACT-B questionnaires before the start of radiotherapy, at the end of radiotherapy and then at 6, 12 and 18 months. While various QoL endpoints are of interest, the primary QoL endpoint of interest will be the summary score of EORTC QLQ C30 derived from 15 questions as defined by Giesinger et al. [27]. We will be comparing the proportion of patients in whom the summary score of the EORTC QLQ C30 is equal to or better than the baseline at 12 months in the two arms.

### Participant timeline {13}

The participant timeline for treatment and assessments is shown in the table below (Table 3).

**Table 3:** Participant timeline

Assessment	All follow-up time points are taken from the date of completion of RT					
	Baseline	Adjuvant RT			3–12 months every 3 months after RT	13–60 months every 6 months after RT
		Week 1	Week 2	Week 3		
Clinical evaluation	X					
Eligibility checklist	X					
Informed consent	X					
Randomization	X					
RT QA	Prior to centre initiation and throughout the trial recruitment period					
RT treatment (control arm)		X	X	X		
RT treatment (test arm)		X				
RT verification (Control Arm)		X	X	X		
RT verification (test arm)		X				
SAE		X	X	X	X	X
Acute toxicity		X	X	X		
FU assessments				X	X	X
EORTC QLQ C30 (control arm)	X			X	At 6, 12 and 18 months after completion of RT	
FACT-B (control arm)	X			X	At 6, 12 and 18 months after completion of RT	
EORTC QLQ C30 (experimental arm)	X	X			At 6, 12 and 18 months after completion of RT	
FACT-B (experimental arm)	X	X			At 6, 12 and 18 months after completion of RT	

Note that patients who undergo sequential tumour bed boost will have their post-radiotherapy assessment postponed till boost is completed. This would mean at the 2nd week in patients receiving 1-week RT and the 4th week in patients undergoing 3-week RT

In each study visit, a clinical assessment including a history and examination will be undertaken. Adverse events related to radiotherapy will be recorded in a case record form. Quality of life questionnaires will be administered on the visits as indicated. During the follow-up period patients will undergo mammogram of intact breast(s) at 12–18-month intervals or as per the institutional policy.

### Sample size {14}

#### Non-inferiority margin and justification for the same

Assuming that the locoregional recurrence rate in the control arm is 5%, a non-inferiority margin of 3% is clinically acceptable and corresponds to a hazard rate of 1.63. The choice of the non-inferiority marginis arbitrary to a significant extent and we base it on the guidance that it should not be larger than the expected benefit from adjuvant radiotherapy. A large volume of data is available regarding the benefit of adjuvant radiotherapy in breast cancer patients. In the setting of breast conservation surgery, the EBCTCG meta-analysis demonstrated that use of adjuvant radiotherapy reduced the 5-year local recurrences by 17% after breast conservation surgery [2], while in the postmastectomy setting the 5-year local recurrence was reduced by 14.7% for N+ patients [3]. The START trials were designed to show a difference of 5% as the non-inferiority margin [28]. This is approximately 33% of the benefit that can be expected from RT vs no RT. Since the START trials have demonstrated that the 40 Gy is non-inferior to 50 Gy we can thus consider a non-inferiority margin of 3% as appropriate for this study.

#### Sample size

The sample is calculated based on the assumption that the 5-year local recurrence rate in the control arm is 5% with an exponential distribution. We hypothesize that the use of 1-week course of adjuvant radiotherapy will not increase the locoregional recurrence beyond 8% (absolute difference of 3%), corresponding to a hazard ratio of 1.63. We further assume that patients will be accrued over 5 years with an initial ramp-up in the first year and that 2% of the patients will be lost to follow-up each year of the trial. The total trial duration is expected to be 10 years to ensure a minimum follow-up of 5 years for the last patient. For a fixed sample size design to achieve the required number of events to achieve a one-sided type I error of 0.025 and a power of 80% the total number of events required is 140. The total planned sample size is 2100 patients.

### Recruitment {15}

Patients will be recruited from the outpatient departments of the disease management group at each institute. No advertising is permitted for recruitment and no inducements will be given for the recruitment of patients in the study. Central randomization will be done at Tata Medical Center using permuted block randomization.

## Assignment of interventions: allocation

### Sequence generation {16a}

Stratified randomization using permuted blocks will be used for randomization. The randomization sequence will be generated using the Robust Randomization App (available at https://clinicalresearch-apps.shinyapps.io/rrapp/) or a similar application and the randomization scheme generated from the app will be then used with the RedCap Randomization module to generate the randomization sequence. Patients will be allocated into the two groups in a 1:1 ratio. The following stratification factors will be used:

1. Type of surgery: mastectomy or breast conservation
2. Regional nodal radiation: required or not
3. Triple-negative breast cancer: yes or no
4. Institute

### Concealment mechanism {16b}

Randomization of the patient will be done using the randomization module implemented in the RedCap randomization module in the study database. Central randomization will be done to ensure allocation concealment.

### Implementation {16c}

After screening and eligibility checks are completed, consenting patients will be randomized using the randomization module. The randomization will be done centrally at Tata Medical Center. RedCap provides a data access group feature which ensures that randomization is stratified by the institute. After filling the other stratification factors, the randomize button will be clicked which will then provide the arm allocation information. This information is permanently recorded in the database and cannot be altered in future.

## Assignment of interventions: blinding

### Who will be blinded {17a}

Given the nature of intervention used in the study, no blinding is planned in this study.

### Procedure for unblinding if needed {17b}

As blinding is not being done in this trial, no unblinding will be required.

## Data collection and management

### Plans for assessment and collection of outcomes {18a}

Data related to the trial will be collected and maintained on a RedCap database maintained in Tata Medical Center. The randomization module of RedCap will be used for random allocation into the two arms. Participating institutes will have access to the RedCap data entry system. The RedCap data forms can be printed out and used as such for clinical data entry in the trial if paper form entry is needed. The following forms will be created in Redcap for the data collection purpose:

1. Demographic information
2. Trial screening form
3. Pretreatment assessment
4. Radiotherapy plan details
5. Radiotherapy plan QA details
6. Radiotherapy treatment setup inaccuracy data
7. Radiotherapy treatment review form
8. Follow-up review form
9. Outcome form
10. QoL capture form
11. SAE reporting form

The RedCap features for longitudinal data collection as well as repeating forms will be utilized for data collection. RedCap also has the facility to set up rules for data quality assurance which will be used to ensure that entered data is of high quality. Planning CT and treatment plan data will be archived in an image bank which is concurrently undergoing development in our institute [29].

### Plans to promote participant retention and complete follow-up {18b}

If a patient or investigator decides to stop the study treatment then the patient’s health status will be periodically reviewed via continued study visits or phone contact, or from their general practitioner or medical records to allow the collection of outcome data. Follow-up assessments including completion of the quality of life questionnaires should still be completed if the patient is willing.

In the event that a patient withdraws from the study entirely, the effective date of the notification will be the date on which their withdrawal is received by the study team. No information about the patient will be collected from that point in time onwards but any information collected prior to that date can be used and forms part of this study.

For patients moving from the area during follow up, every effort should be made for the patient to be followed up at another participating trial centre and for that trial centre to take over responsibility for the patient. A copy of the patient Case Record Forms (CRF) will need to be provided to the new site after appropriate patient consent. Until the new centre agrees (in writing) to take over responsibility, the patient remains the responsibility of the original centre.

### Data management {19}

Access to the data will be available to the principal investigator of the study as well as to the co-investigators after the completion of the study. The DSMC and trial statistician will have access to data while it is ongoing. The data quality and integrity will be checked at quarterly intervals using the data quality checking system available in Redcap. Patient identifiers will be noted as such in the Redcap database and the same will be used for ensuring that any data exports contain de-identified data only. Institutes will have access to the data of their own institute during the trial phase. Once the trial is completed, the main database will be checked for data consistency and quality and then closed for analysis. Additionally, the PI will be asked to sign off key CRFs electronically to ensure completeness and accuracy of the data.

Source documents pertaining to the trial must be maintained by investigational sites. Source documents may include a subject’s medical records, hospital charts, clinic charts, the investigator’s subject study files and the results of diagnostic tests such as X-rays, laboratory tests, and electrocardiograms. The investigator’s copy of the case report forms serves as part of the investigator’s record of a subject’s study-related data. All study-related documentation will be maintained for 10 years following completion of the study or according to existing regulatory requirements.

The following information should be entered into the subject’s medical record:

1. Subject’s name, contact information and protocol identification.
2. The date that the subject entered the study and the subject number.
3. A statement that informed consent was obtained (including the date).
4. Relevant medical history
5. Dates of all subject visits and results of key trial parameters.
6. Occurrence and status of any adverse events.
7. The date the subject exited the study, and a notation as to whether the subject completed the study or reason for discontinuation

### Confidentiality {27}

The study will be conducted in accordance with applicable rules and regulations. All data generated in this study will remain confidential. All information will be stored securely at the sponsoring institute and will only be available to staff directly involved with the study. For documents transferred between institutions, the data will be securely stored in the RedCap database and access restricted to the trial institute and the CTU through the use of the data access groups features in RedCap.

### Plans for collection, laboratory evaluation and storage of biological specimens for genetic or molecular analysis in this trial/future use {33}

Blood will be collected at the beginning and end of radiotherapy for consenting patients. Ten milligrammes of venous blood will be collected and stored in a Ethylene Diamine Tetraacetate (EDTA) blood tube. The sample will be processed for storing the plasma and buffy coat separately in the biobank. Future studies will be designed to utilize these biospecimens for correlative translational studies investigating fraction sensitivity.

## Statistical methods

### Statistical methods for primary and secondary outcomes {20a}

The experimental arm will be compared against the control arm for all primary analyses.

As this is a non-inferiority trial, Kaplan-Meier estimates of 5-year locoregional recurrence incidence rates will be reported [30]. Cumulative incidence of 5-year locoregional recurrence and invasive disease recurrence will also be estimated using cumulative incidence function (with death being considered as a competing event). Cumulative proportions of time to event endpoints like overall survival and invasive disease-free survival will be reported using Kaplan-Meier method. The hazard ratio and the 95% confidence intervals for the locoregional recurrence rate will be computed using the Cox regression analysis. The upper bound of the 95% confidence intervals of the hazard ratio would be used to demonstrate non-inferiority for locoregional recurrence which would be concluded if the same exceeds the predefined critical threshold of 1.63. Exploratory confirmatory competing risk analysis will be done for ipsilateral locoregional recurrence using a Fine-Gray competing risk model (death considered as the competing endpoint). The chi-square test with continuity correction will be used for comparing the binary/categorical variables, while the Wilcoxon test will be used for comparing continuous variables.

All tests will be two-sided and a significance level of 5% will be used for declaring statistical significance. The proportion of patients experiencing any grade 3–4 AE will be reported in each arm and compared between the two arms using the chi-square test or Fisher’s exact test. Similar comparisons will be done for each AE term between the two groups. Comparison of AE between the two arms will be done on full analysis set as well as the per-protocol set.

Multivariable analysis for the primary and secondary endpoints will be conducted using Cox regression analysis. For every model, the underlying assumptions for the model will be checked which would include checks for proportional hazards assumptions, outlier and overly influential detection as well as a check for the linearity assumptions. Additionally bootstrapping will be used to estimate the calibration and discrimination of the model. Further details can be found in the statistical analysis plan.

### Interim analyses {21b}

An interim analysis will be planned once 50 patients are accrued in each arm, and in this analysis, we will be evaluating the acute toxicity of the two interventions. The sample size of 50 patients in each arm allows us 80% power to exclude a within-group rate of CTCAE Grade 3 or more acute skin toxicity exceeding 10% (absolute increase) over the target rate of 5% (one-sided type I error of 5%).

The next interim analysis for safety (in terms of locoregional recurrences) will be planned at 3 years when 16 events in total are expected to have occurred. As a result of this interim analysis, the sample size has been increased to 2100 patients (from 2074 without the interim analysis). The critical *Z* value at the time of interim analysis is 3.47 for futility. The following table illustrates the key features of the design (Table 4).

**Table 4:** Tabulation of the features of the interim analysis for loco-regional recurrence

Analysis	Value	Efficacy	Futility
**IA1: 11%**	*Z*	3.47	− 1.48
*N*: 1184	*p* (1-sided)	0.0003	0.9318
Events: 16	HR at bound	0.28	3.47
Year: 3	*P* (cross) if HR = 1.63	0.0003	0.07
	*P* (cross) if HR = 1	0.0056	0.008
**Final**	*Z*	1.96	1.96
*N*: 2100	*p* (1-sided)	0.025	0.025
Events: 140	HR at bound	1.17	1.17
Year: 10	*P* (cross) if HR = 1.63	0.025	0.975
	*P* (cross) if HR = 1	0.800	0.200

Asymmetric two-sided group sequential design with binding futility bound, 2 analyses, time-to-event outcome with sample size 2100 and 140 events required, 80% power, 2.5% (1-sided) type I error to detect a hazard ratio of 1 with a null hypothesis hazard ratio of 1.63. Enrollment and total study durations are assumed to be 5 and 10 years, respectively. Efficacy bounds derived using a Lan-DeMets O’Brien-Fleming approximation spending function. Futility bounds derived using a Lan-DeMets O’Brien-Fleming approximation spending function. 1A1 11% = 1st interim analysis done when 11% of the local recurrence events have been observed (information fraction)

### Methods for additional analyses (e.g. subgroup analyses) {20b}

The following pre-specified subgroup analyses will be conducted on the primary and the other time to event endpoints stratified by:

1. Menopausal status: premenopausal vs postmenopausal
2. Nodes positive: none vs 1–3 vs > 3
3. Subtype: luminal vs Her2-enriched vs TNBC
4. Regional nodal radiation: none vs SCF alone vs IMN + SCF
5. Boost type: SIB vs sequential boost

These subgroups are chosen as they have an implication on the primary as well as the secondary time to event outcomes and are independently prognostic. Results will be presented as forest plots with interactions results alongside. The interaction test will test if the treatment effect is modified by the subgroup.

In order to evaluate the impact of prognostic factors on the treatment effect, a nomogram will be built using Cox regression that includes treatment as a factor. The use of this nomogram will enable the user to judge the benefit arising from the use of a particular fractionation regimen in a patient with a given combination of prognostic factors.

### Methods in analysis to handle protocol non-adherence and any statistical methods to handle missing data {20c}

For time to event endpoints, we will censor the patient at the date of last follow-up unless the patient is documented to have an event. We will attempt to contact the patients telephonically or by other methods to ensure adherence to follow up. If more than 10% of the patients have missing data, then an additional sensitivity analysis will be undertaken where all lost to follow up patients would be assumed to have the event of interest. For quality of life data, we will under complete case analysis and as a sensitivity analysis missing values will be imputed using an imputation based approach.

Finally, as this is a non-inferiority trial, if there is high non-compliance with the test treatment, then an analysis of only those receiving per-protocol treatment will be conducted separately.

### Plans to give access to the full protocol, participant level-data and statistical code {31c}

The full protocol as well as the statistical analysis plan is available as supplementary material with the manuscript. At present, there are no plans to make patient-level data available publically, but we will consider the same once primary reporting of the outcomes is completed or as mandated by regulations.

## Oversight and monitoring

### Composition of the coordinating centre and trial steering committee {5d}

The trial will be managed through the CTU at the Tata Medical Center. All PIs of the collaborating institutes will be a part of the trial management committee. Study coordination, monitoring, data acquisition and management and statistical analysis will be performed by Tata Medical Center.

Additionally, we will constitute an outcome assessment committee who will provide an independent and central assessment of the important trial outcomes.

### Composition of the data monitoring committee, its role and reporting structure {21a}

An independent data safety and monitoring committee will be constituted which will provide an independent assessment of the patient safety, trial progress, making recommendations to the trial management committee about the continuation of the trial based on the data made available by the trial investigators. The DSMC will also recommend any revisions in protocol required as a result of new or emerging information regarding the adjuvant radiotherapy schedule for this setting. DSMC reports will be communicated to the institutional review boards of the participating centres. The DSMC will be composed of four to five members of whom at least one will be a statistician with significant experience in clinical trials.

### Adverse event reporting and harms {22}

All adverse events related to radiotherapy as well as any serious adverse events will be recorded in clinical trial forms which will be recorded in the centrally managed RedCap database. The investigator at each site will be responsible for reporting the SAE to the respective Institutional Review Board as well as the PI at the CTU. Local IRB would be notified of any SAE within 24 h on a working day or as per the institutional norm. The sponsor will be intimated within a period of 10 days from the date of occurrence of the SAE. SAEs must be reported up to 30 days from the end of the study intervention.

The following information should be provided for all SAEs:

1. Event description including classification according to NCI CTCAE
2. Primary and secondary diagnosis of the event (if death/hospitalization)
3. Severity/worst grade
4. Attribution to study intervention
5. Expectedness (listed in IB/product information)
6. Action taken with the study intervention
7. Impact of SAE (e.g. hospitalization details)
8. The outcome of SAE including end date if recovered

### Frequency and plans for auditing trial conduct {23}

Compliance to the protocol will be reviewed after accrual of the first 5, 25, 75 and 200 cases. Any protocol deviation needs to be notified to the respective IRB, to the chief investigator, coordinating centre and discussed in the trial management committee meetings. Additionally, PI at each centre should be notified about any changes made to the protocol in the interest of patient safety as a result of this—this will need updating in writing in the trial protocol with a change in the version number of the study protocol. Any major radiation dosimetric protocol deviation in more than 1 patient of the first 5 patients or more than 10% cases after 10 have been recruited, should be informed and discussed in the trial management committee. Additional audits and monitoring as required by trial sponsors would be done as mandated by institutional and regulatory norms.

### Plans for communicating important protocol amendments to relevant parties (e.g. trial participants, ethical committees) {25}

Changes and amendments to the protocol would only be made by the Trial Management Committee. Approval of amendments by the Institutional review board (IRB) would be required prior to their implementation. In some instances, an amendment may require a change to a consent form. In such cases, the form will be used only after approval from the IRB. All amendments will be stored in the sponsor site and will be shared with all participating sites through email as well as during regular trial management committee meetings.

### Dissemination plans {31a}

A clinical study report which summarizes and interprets all the pertinent study data collected will be issued which may form the basis of a manuscript intended for publication. The clinical study report or summary thereof will be provided to the local IRB of the institutes participating in the protocol. Additionally the same will be made available on the trial registration websites. The Trial Management Committee will appoint a Writing Committee to draft manuscript(s) based on the trial data. Manuscript(s) will be submitted to peer-reviewed journal(s). The Writing Committee will develop a publication plan, including authorship, target journals and expected dates of publication.

## Discussion

The radiobiological equivalence of the regimen employed in the study is depicted in the table below. For patients requiring regional nodal irradiation, the dose as given in the mastectomy columns applies for the regional nodal volumes, as SIB will not be used in regional nodal irradiation. As demonstrated in the table, the proposed doses are gentler for the normal tissues in the regional nodal volumes (Table 5).

**Table 5:** Table showing the radiobiological calculations for the biologically equivalent doses (BED) of dose schedules to be used for the trial assuming different scenarios for the α/β ratio for breast tumour

Parameters	Mastectomy		Breast conservation (with SIB)	
	Standard	Experimental	Standard	Experimental
Dose (Gy)	40	26	48	32
Dose per fraction	2.67	5.2	3.20	6.4
BED (Gy_2.5_)	82.7	80.8	109.4	113.9
BED (Gy_3_)	75.6	71.1	99.2	100.3
BED (Gy_3.5_)	70.5	64.6	91.9	90.5
BED (Gy_4_)	66.7	59.8	86.4	83.2
For brachial plexus only (SIB used only for breast tumour bed—not for SCF)				
BED (Gy_2_)	93.3	93.6	NA	NA
BED (Gy_1.5_)	111.11	116.13	NA	NA

Note that for normal tissue reactions, the α/β ratio is more well defined at 3–3.5. Gy3, Gy3.5 and Gy 4 represent the BED calculations assuming the α/β ratio of 3, 3.5 and 4, respectively. The dose calculations for breast conservation are for the volume receiving SIB. If a sequential boost of 12 Gy in 4 fractions is given, then the BED values are 99.6 Gy_3_, 92.8 Gy_3.5_ and 87.7 Gy_4_

Dosimetrically, the use of an IMRT SIB is desirable as it reduces the volume of breast exposed to higher doses per fraction. This is a particular concern when hypofractionated radiotherapy regimens are used due to the so-called triple-trouble, where the higher dose, higher dose per fraction and larger volume exposed to the said dose combine to increase the risk of late toxicity. However, IMRT SIB not only increases the delivery time if fixed fields are used but also increases the exposure of the normal tissues to low doses of radiotherapy. Hence, the study will be using a VMAT-based SIB approach where reduction of normal tissue doses to organs at risk is prioritized. A class solution for the VMAT SIB technique has been developed in our institute and has been reported separately. As shown in the report, our SIB technique results in better conformity while maintaining the low dose sparing for the normal organs and also reduces the MU requirements.

Use of a 1-week radiotherapy course is likely to be substantially resource sparing if efficacy is equivalent. If radiotherapy is completed in 1 week, instead of 3 weeks, an indirect cost saving of approximately 66% is expected. Additionally, machine time will be significantly spared allowing three patients to be treated in the same treatment slot improving the accessibility of radiotherapy significantly. A separate substudy will look at the cost-effectiveness of the technique.

A large multi-centric randomized controlled trial poses unique challenges in our setting. While the number of eligible patients is large, a significant number of patients face issues in maintaining follow-up for a long duration of time as they often present from far-flung areas. Institutional practices are variable and availability of planning techniques and equipment is not equitable. As most of the health care expenditure is out of pocket, patients are often unable to afford the latest systemic therapy regimens. Hence, results obtained in Western trials cannot be generalized for our nation. As a part of this study, we also hope to standardize breast cancer radiotherapy delivery in India and integrate image-guided radiotherapy in major cancer centres across the nation.

In summary, the study aims to investigate a shorter treatment schedule offering a more accessible essential treatment option in patients with breast cancer. Although five fraction regimes have been tested in the FAST and FAST-Forward study [18, 31], our study tests a few novel elements to complement this research. This include (a) testing the efficacy and toxicities of a five fraction adjuvant radiation therapy regime in a population with higher percentage of node positive disease, (b) simultaneous integrated boost to the tumour bed and internal mammary chain nodes in 5 fractions and (c) validating five fraction RT in a non-Caucasian population.

## Trial status

The HYPORT-Adjuvant trial was IRB approved and current protocol version as of 7th June 2020 is version 6.0 (dated 8th July 2019). The trial was prospectively registered in the Clinical Trial Registry of India (CTRI) vide registration number: CTRI/2018/12/016816 (31 December 2018) as well as the ClinicalTrial.gov website at NCT03788213 (28 December 2018). The first patient was randomized on 28 March 2019 and accrual is expected to last for 5 years (tentatively till April 2024). The trial follow-up will be extended for a period of 5 years, and we expect that the events for the primary endpoints will mature by April 2029. The trial is currently accruing patients, and to date, 420 patients have been randomized (7th June 2020).

## Abbreviations

AE: Adverse events
BED: Biologically effective dose
CMC: Christian Medical College
CTCAE: Common Terminology Criteria for Adverse Events
CTRI: Clinical Trial Registry of India
Dmax: Dose maximum
DSMC: Data Safety and Monitoring Committee
EORTC: European Organisation for Research and Treatment of Cancer
FU: Follow-up
Gy: Gray
iDFS: Invasive disease-free survival
IMN: Internal mammary node
IMRT: Intensity modulated radiotherapy
LRR: Locoregional recurrence
MV: Mega-volts
NCI: National Cancer Institute
OS: Overall survival
RCT: Randomized controlled trial
SABCS: San Antonio Breast Cancer Symposium
SAE: Serious adverse events
SCF: Supraclavicular fossa
SGPGI: Sanjay Gandhi Post Graduate Institute of Medical Sciences
SIB: Simultaneous integrated boost
SMART: Simultaneous Modulated Accelerated Radiotherapy
TNBC: Triple-negative breast cancer
VMAT: Volumetric modulated arc therapy
